# Report of the Committee on Practical Medicine

**Published:** 1856-09

**Authors:** Samuel Thompson

**Affiliations:** Albion, Edwards County, Ill.


THE NORTH-WESTERN

### MEDICAL AND SURGICAL JOURNAL.

(NEW SERIES.)

VOL. V. SEPTEMBER, 1856. NO. 9.

### ORIGINAL COMMUNICATIONS.

ARTICLE I.—*Report of the Committee on Practical Medicine.* By SAMUEL THOMPSON, M.D., of Albion, Edwards County, Ill. Presented to the State Medical Society.

[Concluded.]

Pneumonia, as might have been expected, occupied quite a prominent place among the diseases of last winter and spring. Dr. Nance observes, in his letter to the committee, "That pneumonia and broncho-pneumonia have been quite prevalent since the winter broke up. But very few cases of *pleuritis* have been seen in this vicinity for some years." This singular fact, we believe, applies to a large range of country. Dr. Haller, of Vandalia, informs the committee, "that, during the months of January and February, there were quite a number of cases of typhoid pneumonia, which I treated with *alteratives, anodynes, tonics, stimulating expectorants, &c.*, with the auxiliaries, *rubefacients, and vesicants.* I have not bled a single case of pneumonia this season of any kind, but in many I have used the tartar emetic freely, and there has been less fatality than usual." Now we do protest against such lawyer-like generalizations. Here, we request, on behalf of the profession, *facts;* and put a string of categorical questions; and we have for answer, that quite a number of cases occurred, that nearly every class of remedies comprised in the Pharmacopœia were used, and that less than the usual number died: now, even here, unless we know what is the usual mortality in the Doctor's neighborhood, how can we gain even approximative information? Dr. Harris considers, that round Ottawa the number of cases of pneumonia ranked next to those of Periodic fevers, while a great many of the cases bore the intermittent stamp, and not only bore but needed quinine in the treatment.

Dr. Payne writes that early in the spring, pneumonia began to prevail, and has continued, with some abatement, up to the present time, (May 16,) and that the disease has been much more fatal than has ever been known before. Commencing as a bronchitis for several days, when pneumonia, with typhoid symptoms supervened. But we will let him speak for himself. "During the extreme cold weather of the winter we had little sickness. Early in the spring, however, pneumonia prevailed, and has continued, but with some abatement, up to the present time. The disease has been much more fatal than has ever been known before in this locality. In almost every case it has been complicated with early typhoid symptoms, and in some cases so completely masked by the occurrence of typhoid as to render it much more difficult to form a correct diagnosis. In fact, the typhoid symptoms were so prominent that some physicians would have denominated it typhoid fever with pneumonia as a complication. The disease, in a majority of cases, was preceded by a severe cold, in other words, bronchitis, for several days, usually ushered in with a distinct chill, or a chilly sensation, and in a short time, followed by frequent pulse, flushing in the face, pain in the side, usually the left, cough, with expectoration of mucus, with more or less blood. In many cases there was great oppression at the stomach, with nausea and vomiting—tongue with a heavy bilious coating at the commencement. These symptoms, unless arrested, would increase, or assume a different form. The pulse, instead of becoming hard, became more easily compressed, but more frequent—less difficulty of breathing, but more disturbance of the mind, and, in several instances, amounting to complete delirium. The tongue now usually became dry, and in some cases, swelled and red around the edges.

The bowels bound at the commencement, but generally afterwards a tendency to diarrhœa. If the case proved fatal, these symptoms would increase to the ninth, and in some cases, to the twelfth day. I should have remarked that in the majority of cases there was great bilious derangement, a jaundiced state of the skin, and vomiting of large quantities of bilious matter, which would continue for four or five days after the disease was ushered in; so that we had superadded to the typhoid symptoms, bronchial inflammation and a system poisoned as it were with bilious matter. This description is not intended to answer for all, for in some cases the inflammatory symptoms were prominent from the commencement to the termination of the disease. *Congestive pneumonia* occurred in several cases, proving fatal in most of them. I was so fortunate as to see but one case, and that terminated fatally. Reaction was imperfectly established in about 48 hours from the time he was taken with the chill; but the prostration was so great, and the nervous system so overwhelmed by the shock of the disease, that he died on the evening of the eighth day. The treatment of these cases was different. In the first or typhoid form, much active treatment could not be used, on account of the great tendency to prostration. A full dose of calomel and ipecac, if given early, had a good effect; in some cases it was arrested at the threshold, and where this was not the case its violence was moderated. In those cases where the disease was fully formed, and the typhoid symptoms superadded, I preferred giving the calomel in smaller doses, say 12 grains of calomel in three portions, with 5 grains of Dover's powder, with each combined, and this worked off in some twelve or fourteen hours with castor oil, if necessary; expectorants, as comp. syr. of squill, were used in cases where the stomach was not already irritable; diaphoretics were also used after the operation of calomel. I usually give Dover's powder, every two or three hours, in 8 or 10 grain doses, and continued the patient on their use for some 24 or 48 hours, until I again considered it necessary to repeat the calomel. Later in the disease stimulating expectorants, and quinine as a tonic, and where necessary brandy as a stimulant, and a blister over the region of the affected lung after the 2d or 3d day was beneficial. In the second form I have referred to, viz., those cases of an active inflammatory character, depletion was borne to a greater extent. We do not deplete with the lancet; in fact, bleeding in this section in pneumonia, I believe, is entirely abandoned: calomel in active doses, and tartar emetic in nauseating doses, answering all the purposes that might be obtained by the use of the lancet. Later in the disease, syrup of squills and senega, as a stimulating expectorant, is used. But few cases of this kind occurred, and all, so far as I am able to learn, recovered. In the congestive form, the indication was to establish reaction, and, after reaction was established, to promote expectoration and guard against prostration."

"The only disease which has prevailed as an epidemic to any extent, within the last two years, is acute bronchitis. I have already spoken of this disease, in my remarks on pneumonia. Many of the adult population, and particularly the males, were attacked with it; it did not prove serious in any of these cases, most of them being able to attend to their ordinary business; the greatest danger was its liability to terminate in pneumonia. It was, however, among children, of a more aggravated form, usually coming on with a chill, or chilly sensations, followed in a short time by high fever, tightness across the chest and down the sternum, great oppression referred to the epigastrium, severe paroxysmal fits of coughing, the mucus occasionally mixed with blood; there was a remission in most cases in the fever towards morning. The disease was of various grades, from the mildest to the most aggravated form: the duration of the disease was from ten days to two weeks. It made its appearance the latter part of March, continuing for five or six weeks before it declined. I had quite a number of cases under my charge, all of which recovered, and so far as I am able to learn but one or at most two deaths occurred among all the cases. The disease was principally confined to Marshall and its vicintity, but a few cases occurred in the country. It was confined principally to the eastern portion of the town, and where it seized upon one member it was usually followed by others, and in some few cases every member of a family was attacked. There was nothing very novel in the treatment; calomel was required in some cases where there was much bilious derangement, or the inflammatory action ran high. In those cases where there was a marked remission, quinine seemed to be of the greatest benefit, cutting short some few cases and materially moderating the violence of others. There were several cases that I believe would have terminated fatally, had it not been for the use of this valuable agent. All of our physicians, so far as I have conversed, made use of it. Diaphoretics, expectorants, and a blister over the sternum were used." In our own neighborhood (Edwards and Wayne Counties) there has been an almost universal prevalence of bronchial affections, especially during April and the beginning of May, constituting in fact a true influenza, in which the affection of the larynx was the most prominent and troublesome symptom: and we are informed the same state of things extended over the counties adjoining. Our experience in the prevalence of pneumonia, also, fully confirms the statements of Drs. Nance, Haller, Harris, and Payne; for though the cases under our observation have hardly presented quite so markedly typhoid a character as they describe to have been present, yet the number of cases of pneumonic affections have been greater than we remember to have met with for many years in our generally healthy neighborhood. Comparing, for instance, our experience in 1855 with 1856, we find that from January to July of the former year we have notes of but 7 cases of pneumonia proper, and 7 cases of other affections of the respiratory organs, making 14 in all, and no deaths: while, beginning on 20th November, 1855, and ending on 16th May, 1856, we have notes of 55 cases of affections of the respiratory organs, of which 35 were cases of pneumonia, the last of which was on 21st April, and 20 were chiefly of the character of influenza. Of the cases of pneumonia, 6 died—1 in February, 2 in March, and 3 in April. Of the 2 in March, one was a man with tubercles in the lungs, who in November had recovered from pneumonia complicated with intermittent, and who was recovering from the last attack, when, on 7th March, during a very cold wind, he insisted on getting up and going out to urinate—he died the next day:

the other was a lady of scrofulous habit, who had been under a Steamer or Eclectic for some days, and finally became insane, which she had been some years before. Three of the remaining cases died in one house, mother, daughter, and son, from want of protection from the cold wind—the cabin was like a seive. These last were the most truly typhoid cases of pneumonia we have seen this season; but in nearly every instance we remarked the peculiar predominance of bronchitic symptoms in the first stages of nearly every case. Our treatment was generally tartar emetic and opium, according to the following formula: — Tartar emetic, ʒ j.; powdered opium, ʒ ss.; ext. gentian, q.s.; divide into 120 pills. We consider the combination of the extract of gentian highly useful, as somewhat counteracting the debilitating effect of the tartar emetic. Of these pills, either whole or divided, according to circumstances, we are in the habit of giving one every two hours (if the stomach will bear it) while the acute symptoms last; substituting in very typhoid cases, &c., calomel and opium, or calomel and quinine with opium, or ipecac and Dover's powder, according to circumstances. We prefer administering tartar emetic in pill for two reasons: when given in solution or in powders we have frequently found it produced a troublesome irritation of the throat and fauces; and once we met with a case of poisoning by tartar emetic, from a child taking up the cup containing the solution and drinking it for pure water. Blisters in *acute* cases, except in the latter stages, when the febrile symptoms have much abated, we do not like: and we fully concur with Dr. Payne, that bleeding under the present adynamic constitutional condition would be generally improper.

Of the exanthemata there would appear to have been a large number of cases. Dr. McBane, of Metropolis City, reports nine cases of *variola* in one family, occurring from January to April; all recovered, and the disease did not spread. Dr. Nance, of Stark County, also reports that during the past winter variola prevailed extensively in and near the village of Altona, on the C. M. I. Railroad, and also at Walnut Grove, in Knox County, and considers that not less than forty persons were attacked, though very few died; and notwithstanding that the citizens were guilty of great carelessness, general vaccination prevented its spread. The non-extension of the disease in these cases, is a strong argument against the opinions we have seen broached by medical men, both in England and America, as to the inefficiency of vaccination. We wish our correspondents had given us a description of the grade of the disease, the sequelæ, and whether any means (and if any, what means,) were used to prevent the pitting of the skin, &c., &c. As it is, the record of fact of the occurrence of the diseases teaches little for the future.

From Chicago, the only accounts we have obtained, are derived from the *N.-W. Journal*, for April, 1855, wherein Dr. Davis, at the Cook County Medical Society, refers to the unusual prevalence of small-pox, and the rapidity and extent to which it had spread, remarking that he considered it could not be accounted for by direct contagion, but that it must be *aided* at least by an epidemic condition of the atmosphere: and in a note by the editors it is stated, that about the same period variola had appeared in a very large proportion of the principal towns of the North-West.

Dr. McBane reports that scarlatina has been prevailing in the higher localities in his neighborhood, and often proved fatal when mercurially treated. He says, "I have seen and treated many cases successfully by avoiding mercury."

In Stark County, we also learn from Dr. Nance, that rubeola has prevailed more extensively during the latter part of the winter, and in the months of March and April, than at any period since the winter of 1847 and 1848. The epidemic appears to have been quite severe, being usually complicated with pneumonia, and sometimes with congestion of the brain, arachnitis, convulsions, &c., nevertheless few died, except in case of those who were previously laboring under phthisis.

In the neighborhood of Vandalia, we also learn from Dr. Haller, that erysipelas has occurred which has been of a more asthenic character than formerly, and bearing the use of tonics. In his report on the sanitary characteristics of Chicago, in *N.-W. Journal*, for May, 1855, Prof. Davis reports 8 or 10 cases of severe erysipelas. In some of these cases, he administered the tincture of the muriate of iron, giving 20 drops every 4 hours, prescribing at the same time turpentine, quinine, and opium. He states, that of the four cases in which the iron was given, it was successful in three, the other case was not benefited by it. In two cases of cutaneous erysipelas, occurring in children under three years of age, the Chairman of the Committee applied the tinct. iodine (diluted with alcohol so as to adapt it to their tender skins) externally, repeating the application as fast as the color faded, and gave at the same time alterative doses of calomel and pulv. Doveri: both these cases quickly recovered. In four other cases, to which he was called early this year, he administered the tinct. of the muriate of iron, 15 to 20 drops every three hours; applied externally the tinct. iodine, and gave calomel and Dover's powder, or quinine, &c., according to the symptoms, and all recovered.

Two other subjects demand our notice, milksickness and cholera. Of the first we have but one report, which we insert, it is from Dr. Haller. He says, "milksickness does occur in some parts of our county, and is confined to particular localities. We find it on small timbered streams, where the water, during a great part of the grazing season, stands in pools, as there is not enough to run. At these places animals get water, and in such localities as this we find the sloes. Our stock, that keep along the river and in the bottom, are not known to have the sloes. The soil along these little streams, is of a black loamy kind in some places and sandy in others, and as we get out from the creeks we find whitish-yellow clay, common on the post-oak flats. This disease occurs most frequently and is much worse in dry seasons than wet. Last season was uncommonly wet, and I did neither see nor hear of a case occurring in our county: notwithstanding, we had more malarial fever than was ever known before in one season—it occurs from the month of July up to cold weather. I am of opinion it is a mineral poison that does the mischief, and it so affects the animals solids and fluids, that whosoever eats of them becomes affected with animal poison, giving the symptoms we find when called upon to treat them. I am led to this conclusion from what I have seen among animals and men diseased with it. When I

am called to see a man with the sloes, I commence giving him hydg. sub-mur. and opium, and continue it until I produce a slight ptyalism, at the same time open the lower bowels with glysters, then I give a good brisk purgative of croton oil, still keeping up the injections. After his bowels are thoroughly moved, if he needs tonics and his stomach is still irritable, I give a few sedative doses of quinine, and brandy *ad libitum;* after this I give tinct. ferri mur. and iodide potassium, looking out to keep the bowels in a laxative state. I continue the iodide of potassium so long as there is any torpidity of the biliary secretion and debility of the system. In short, my system is just about the same as I use for *painter's colic.* My success has been very good. I always ptyalize, and then give the iodide of potassium till they are entirely recovered."

Now, though we can by no means endorse the Doctor's etiology, pathology, or therapeutics, yet we are pleased to receive and to record descriptions of the localities of this endemic and of its treatment, as some further addition to the statistics of a disease about which, as yet, much prejudice, assumption, and theorizing has existed, but about which, as yet, we possess very few reliable facts. We only regret, as in other diseases, that he and others should assume that everybody means to describe a similar pathological condition by the same name. We are very much inclined to think that it would be wise for us in many cases to follow the example of the sign painter, who painted what he designed for a red cow, but supposing everybody might not come to the same conclusion as to what it did indicate, he wrote under it, *this is a red cow.* Now, we would suggest to our brethren, when *they* write the name of a disease, upon the features of which there is not universal consent, that they reverse the sign painter's course, and paint the lineaments underneath. For ourselves, we can confirm Dr. Haller's observation, of the absence of any cases of milksickness last year, and confirm his opinion, expressed by ourselves and others years ago, that it is always most common after *dry* weather. We were the more pleased to have some contribution to copy on this subject at the present time, in order that our brethren in southern Indiana might see that there is not such an utter want of knowledge of this disease as some of them appear to suppose. We refer to an article by our friend, W. H. Byford, M.D., of Evansville, copied from the *Nashville Journal of Medicine* into the *N.-W. Journal*, of this State, for January, 1856, and headed, "Milksickness, Poison, Tires, &c.," in which the author appears to be ignorant that any persons have ever written on the subject before, or to be aware that there is great diversity of opinion as to its etiology and treatment. And, though we are glad to see another and an able man publish his description of a disease which, like typhoid fever, appears to bear a different character, under one name, in different places; yet we should have liked to have seen that the Doctor, in writing a *Systematic Essay for the Benefit of the Junior Members of the Profession*, should have ascertained what others had done, and have referred to what others had written on the same subject, on which, as yet, all certainly are but inquirers and learners: he would perhaps have found, that, in 1810, the late Professor Drake wrote upon and, in 1841, published a pamphlet on the subject; that, somewhere about the same time, Dr. Seaton, in the White River country, in *Indiana*, published a long article on this disease; that Dr. Barber wrote on the subject as far back as 1809; that, in 1822, Dr. McCall, of Tennessee, published an article on this disease in the *Philadelphia Journal*, which is quoted in the *Cyclopædia of Practical Medicine*, published in London, in 1833; that, in 1848, Dr. S. Thompson published an article on the subject, in the *St. Louis Medical Journal*, for November. In the *N.-W. Medical Journal*, for January, 1850, is the record of a case by Dr. J. M. Logan, of Palestine, Ill.; in the same journal, for May, 1850, in an article on "Malaria, &c.," by Dr. S. Thompson, of Ill., are remarks on the subject, with a description of six cases; that, again, in the same journal, is a paper by Dr. J. J. Lesher, of Mt. Carmel, Ill., on milksickness. That, in the Report of the Committee on Practical Medicine for this Society, in 1851, four pages are occupied with the subject. In the *Western Journal of Medicine*, published in Louisville, in May, 1852, is a long and most valuable article on the same subject, by Prof. L. P. Yandell, perhaps the most compendious paper that has been written, and giving many references to authorities, occupying more than 27 pages; but *Dr. Yandell* had never seen anything which had been written in *this* State on milksickness, or at least does not refer to it. In the same journal, in the same year we think, (we cannot lay our hand on it,) is the Inaugural Thesis of Dr. S. W. Thompson, on Milksickness. In the *N.-W. Journal*, for August, 1853, is a paper of 9 or 10 pages on milksickness, by Dr. Samuel Thompson, of Ill., being an essay read before the Æsculapian Society, and published by order of the Society; in the same journal, for May, 1852, is, also, a short article on the treatment of this disease, by Dr. C. W. Davis. Somewhere about 1844 or 1845, Dr. Solon Borland published a small pamphlet on milksickness, containing some interesting statements. Now, when our humble selves, in 1853, presented a very unpretending paper to this Society, on the "Uses of Turpentine," which they did us the honor to approve, *we* received chastisement with a cat-o'-nine tails from our old friend, Dr. Hall, of Toulon, about every month, for we forget how long—because we had failed to quote *all* that Dr. Copeland had written on the *same* subject; which chastisement we bore (as is our nature) in humble silence, though we were dragged round the country every month, with the terrific warning written on our backs, "Render unto Cæsar the things that are Cæsars," giving us at any rate, if not fame, notoriety. But what shall we say to our neighbor of Evansville, who, from so many contributors to this subject, has not noticed *one* — we transfer him to Dr. Hall.

In answer to our circular questions on this subject, this year, Dr. McBane says there has been no milksickness in his neighborhood. Drs. Payne, Nance, and Vincenz, report no cases around them.

On the subject of Cholera, the last-named gentleman relates, that, in June, 1854, a Swiss, lately emigrated, died of cholera. An American family, in the same house, father, son, and daughter, then died of the same disease in two days; that, not very far from this house, an elderly woman, and a young man and his wife died. In a house opposite to this, another man and his little boy died; and in the next house to this a young woman died. Dr. V. did not attend them, and does not know what treatment was used, neither does he state *what was the sanitary condition of the houses and their surroundings;* but in a previous part of his letter he says, there are no swamps or standing waters nearer than 4 miles in a S.W. direction; the village contains about 800 inhabitants, and the country is well cleared. Dr. Nance states that cholera made its appearance about 10 miles distant from Lafayette, in July, 1854; but that since then, till August last, there had been no more cases: then it commenced in a Swedish family, 3 miles from town; but he remarks that the filth *about* the house, with a hog wallow under it, were sufficient to account for any pestilence. Dr. Haller states, that, in May, 1855, there occurred 7 cases of cholera among the railroad hands, 5 of whom died, but that no case occurred among the citizens. And Dr. Davis, in the *Journal*, for Nov., 1855, shows that, from careful meteorological observations it appears, that in Chicago, heat and damp, with a S. or S.W. wind, develop cholera in those pre-disposed, while the *same* conditions, aided by sudden and frequent alterations of temperature, develop inflammatory dysentery. That, in 1855, cholera cases were imported as freely and as surely as in 1854; that, in the last-named year, the meteoric conditions above-named existed, and cholera made fearful ravages; while, in 1855, with similar importations, but different meteoric conditions, it did not spread. We would here express our cordial approbation of his observations on what has always appeared to us nearly a waste of time and paper; we refer to "*mean* temperatures," &c. He truly says, "Observers have been too content with obtaining of mean or average results for given periods of time, without patiently observing the daily variations and coincidences. It is very easy to perceive, that the same month, in two successive years, may give very nearly the same *mean* both of temperature and atmospheric moisture; and yet these elements may be conbined variously in different parts of that time." We would recommend that the above paper be appended to this Report, as a most valuable accumulation of carefully-observed facts in relation to this subject. We also beg to append the whole letter of Dr. Nance, the topographical details, &c., of which are worthy of record, but are too copious to copy.

We have read with much pleasure in the *N.-W. Journal*, for January, 1856, Dr. Davis' very interesting article on the Preservation of Milk, and, though not applying very much at present to our wants in this State, it is of importance to us as emanating from one of ourselves, and also as showing that there really exists, at times, in the cities of the East, &c., a real *bona fide* milksickness, really caused by the ingestion of milk. Also, in the February number of the same journal are two very interesting cases, by Dr. Woodward, of Sharon, Ill. The first, a case of what we suppose might be termed traumatic pneumonia, is almost unique in its details. A piece of hickory nutshell passed down the trachea, and must evidently have entered the bronchia and wounded the lungs by its sharp, ragged edges. Pneumonia and symptoms of pleurisy supervened: every means of relief failed, when, on the 10th day, while apparently in the jaws of death, a violent fit of coughing came on, rupturing an abscess in the lungs, we presume, from which poured forth a stream of pus; and, after again sinking apparently beyond recovery, a second fit of coughing occurred, during which more pus and a piece of hickory nutshell, about $\frac{1}{4}$ inch long and $\frac{1}{8}$ inch wide, was expelled, with more pus. She continued in a very precarious state till the 33d day, when an abscess began to point on the right of the sternum, towards the bottom, which was evacuated by a trocar—she slowly recovered. Was the abscess by the sternum an empyema or a collection of pus in the cellular tissue about the mediastinum? By the same author, in the same number of the journal, is a very instructive case, illustrating the value of Prof. Brainard's suggestions and experiments on the uses of iodine as a remedy for snake poison. Dr. Woodward is so impressed with its potency, as an antidote to animal poison, that he proposes to institute a series of experiments with it as a cure for hydrophobia.

In the March number of the *N.-W. Journal*, we find a very able address, delivered before the Æsculapian Society, by our old and valued friend, Dr. Washburn. We are not aware that its subjects come within the scope of our duties, other than as a paper creditable to our profession in this State, and deserving of serious consideration by us all. It assumes for our profession a high and noble standing, claiming high and noble conduct on the part of its members. It maintains the expediency of legislative enactment to protect the public against the fraud of quackery. And this is a subject on which many of our wisest members can hardly see the right course to take. Any action should be wisely and well weighed before entered upon. It appears to us that while the fact may not militate against the Doctor's views, that the grand fault of the age is *over-legislation*. To one point of Dr. W.'s address we would earnestly direct the attention of our brethren. It is his advice to *ourselves*, his exhortation to put away from among us the carping, jealous listlessness, which has made the ill-will of Doctors towards each other a proverb.

We are half tempted to step out of our department of practical medicine to make some comments upon Dr. Freer's admirable and compendious article upon injuries of joints, and of the elbow-joint in particular, in the April number of the *N.-W. Journal*, but we forbear. Our Report, though deficient of materials, is already extended, we fear, beyond the patience of the Society, and we will simply call attention to the very interesting paper of Dr. Goodbrake, of Clinton, recording the anomalous circumstance of a delivery of the placenta before the birth of the child; and yet that, nevertheless, the child lived. We presume this last fact was solely owing to the promptitude of the Doctor in introducing his finger into the mouth of the child before it was born, thus admitting air into the lungs. We always consider this precaution should never be omitted at the earliest possible moment after the rupture of the membranes. By Dr. Adams, also of Clinton, and in the same number of the *Journal*, we find a record of one of those strange freaks of nature, a monstrosity, an arrest of development, it appears, of certain bones and of other portions of the body.

Dr. Nance, speaking of pneumonia, remarks upon the non-occurrence of pleuritis. It occurs to us that another disease, with which, 8 or 10 years ago, we used to be much troubled, viz., scabies, has not presented itself to our notice for several years.

We have omitted to notice one well-written and instructive article in the *N.-W. Journal*, for March last, by Dr. J. W. McKinney, of New Albany, Ill., on the treatment of Meningitis, with a table of the results of treatment in 29 cases, also showing that it is in the five coldest months of the year that a very large proportion of such cases occur. The Doctor advocates V.S. as the sheet anchor; where this cannot be used, he depends upon calomel, ice, blisters, quinine, &c. Now, it appears to us, that while the energetic use of the lancet must be needful, and calomel, &c., are often indicated; yet that the Doctor overlooked the powerful sedative agency of tartarized antimony, which we think should have played an important part in the treatment, at any rate in cases where the lancet could not be used. *We* should, we think, have even ventured on the combination of tartar emetic, digitalis, and morphia, especially *after* bleeding, perhaps thereby saving much blood; while we confess we have great doubts of the propriety of blisters on any part of the head during the acute stage of a cerebral meningitis. Again, we suspect, that, in the case of Mr. J. F., the frequency and smallness of the pulse *in connection with all* the *other* symptoms did not justify the postponing V.S. till after the operation of an emeto-cathartic. We suspect the pulse would have *risen* with the flow of blood, and thus valuable time have been spared. The Doctor must pardon our criticisms, his experience has evidently been much greater in such cases than ours; for we presume 10 acute idiopathic cases of cerebral meningitis have not occurred in our practice in five years—another singular illustration of how even the acute phlegmasiæ may be endemics.

With these remarks we will draw to a close our voluminous report, with an earnest exhortation to the profession of our noble State, to rouse themselves from their lethargy; to consider, that upon *each one's individual daily* exertions and observations must be built up the dignity and wisdom of our profession; and a hope that every one of us will, from this day forth, note at the bedside the symptoms and treatment of every case we attend: and though many such cases may present nothing very important, the importance can never be judged of beforehand; and though *one* may be valueless, *many* may point to some truth or manifest some error—that we may thus have materials from which to supply facts for our committees to report on, and cases and essays illustrated by facts, for our medical journals to publish.

